# Comparative molecular analysis of colorectal cancer in Saudi Arabia: distinct genetic alterations and clinical features

**DOI:** 10.3389/fonc.2026.1792016

**Published:** 2026-05-01

**Authors:** Maha-Hamadien Abdulla, Jumanah Yousef Alshenaifi, Mansoor-Ali Vaali-Mohammed, Ahmad Zubaidi, Khayal Al Khayal, Noura S. Alhassan, Khalid Abdulrahman Al-Saleh, Omar Al Obeed, Scott Kopetz

**Affiliations:** 1 Department of Surgery, College of Medicine, King Saud University, Riyadh, Saudi Arabia; 2 Department of Pathology, College of medicine, King Saud University, Riyadh, Saudi Arabia; 3 Department of Medicine, Oncology Center, College of Medicine, King Saud University, Riyadh, Saudi Arabia; 4 Department of Gastrointestinal Medical Oncology, Division of Cancer Medicine, The University of Texas MD Anderson Cancer Center, Houston, TX, United States

**Keywords:** colorectal cancer, precision oncology, Saudi Arabia, Saudi cohort, whole-exome sequencing, KRAS, TP53, CRC

## Abstract

**Purpose:**

Globally, colorectal cancer (CRC) stands as the third most common type of cancer, a ranking that has remained consistent over recent years. However, the scenario diverges in Saudi Arabia, where CRC ascends to the forefront as the most common cancer among males and the second most prevalent among females. This discrepancy is primarily driven by male demographics, with a staggering 73% of cases diagnosed at a late stage, underscoring a critical public health concern. Delving into the molecular and phenotypic profiles of CRC within the Saudi populace emerges as a crucial stride towards addressing the escalating disease burden afflicting the country.

**Methods:**

To unravel the CRC driver molecular landscapes among the Saudi population, we performed Whole Exome Sequencing (WES) of 50 Saudi CRC patients collected by the Colorectal Cancer Research Center at King Saud University Medical Center (KSUMC) with MSS adenocarcinoma tumors. These patients were compared with comparable tumor cohorts from MD Anderson Cancer Center (MDACC) and The Cancer Genome Atlas (TCGA).

**Results:**

Tumors from Saudi patients have a significantly lower frequency of *KRAS* mutations (6.5% vs. 45.0% MDACC and 41.1% TCGA, with a p-value < 0.001), *APC* mutations (47.8% vs. 77.9% MDACC and 79% TCGA, with a p-value of less than 0.001) and *CTNNB1* mutations (0% vs. 2.3% MDACC and 10% TCGA, with a p-value < 0.001). However, *TP53* mutations were more common (50.0% vs. 35.9% MDACC and 43.0% TCGA, p-value < 0.05). The proportion of Saudi CRC patients who have sigmoid disease is higher (41.7% vs. 24.4% at MDACC) and they tend to be significantly younger (58 vs. 62 $\tilde{x}$ MDACC and 68 $\tilde{x}$ TCGA).

**Conclusion:**

The results indicate a distinct mutational landscape of CRC in Saudi Arabia, highlighting the importance of gathering population-specific data in order to inform diagnostic of molecular cancer screening and treatment strategies for patients in Saudi Arabia.

## Introduction

Colorectal cancer (CRC) is consistently ranked as the third most prevalent form of cancer and the second most common cause of cancer-related mortality worldwide (1). This statistic starkly contrasts with the epidemiological profile in the Kingdom of Saudi Arabia (KSA), where CRC not only leads as the primary cancer among males but also ranks as the second most common among females and all genders (1–3). This cancer is also the leading cause of cancer-related mortality in KSA (1). Particularly alarming is the high prevalence of late-stage diagnoses, with approximately 73% of CRC cases identified at an advanced stage, which considerably worsens the prognosis (4, 5). These patterns underscore CRC as a major public health issue and a substantial burden for the Saudi healthcare system.

Particularly predisposed to tumors of the left colon and rectum, the early onset of CRC in the Saudi population further differentiates it from patterns observed in other international populations (5–7). Studies suggest that these unique clinical presentations may be linked to specific lifestyles, dietary habits, and genetic factors prevalent in the region (8, 9).

Despite the growing incidence of CRC in KSA, there is a significant gap in the understanding of its molecular underpinnings. Preliminary molecular profiling studies have identified a distinct prevalence of CRC driver genes within the Saudi patients (10–13). These genes include driver genes such as *APC*, *KRAS*, and *TP53*, as well as variants in key CRC pathways. These genes are implicated globally in CRC pathogenesis but exhibit unique mutation spectrums in Saudi patients (10–13). For instance, the mutation rates in the *KRAS* gene among Saudi CRC patients have been reported differently from those in Western populations, which may influence both the disease course and the response to therapy (12).

These previous studies have predominantly employed targeted sequencing and PCR techniques, focusing on mixed patient groups that include both hereditary and sporadic CRC cases. This approach has often clouded the interpretation of data due to the variability in microsatellite stability, tumor mutation burden, and the presence of germline mutations—factors that are critically important in determining the molecular profile and prognosis of the tumor.

In light of these unique factors, our study introduces the first comprehensive whole-exome sequencing (WES) analysis focused exclusively on sporadic microsatellite stable (MSS) CRC patients in Saudi Arabia. By isolating this particular group, our research aims to delineate the full spectrum of genetic alterations that drive CRC, free from the confounding effects of mixed patient data. This targeted approach not only promises to enhance the specificity of our findings but also amplifies the potential translational impact of our study, proposing novel genetic markers that could guide personalized treatment strategies tailored to the genetic makeup of Saudi CRC patients.

The nation’s comparatively conservative genetic pool, characterized by elevated consanguinity rates, presents a distinctive opportunity to examine CRC through the perspective of genetic predispositions that may markedly differ from those found in more genetically diverse populations. Additionally, understanding the molecular characteristics of CRC in the Saudi population is inadequate, resulting in a lack of knowledge that is increasingly crucial when it comes to predicting prognosis and selecting suitable therapies.

## Methods

### Cohorts

This study enrolled 48 patients with colorectal cancer at the colorectal cancer research center at King Saudi University Medical City (KSUMC). The patients’ samples and clinical data were stored in the center’s established biobank system. We selected patients based on the following criteria: i) the tumor was an adenocarcinoma; ii) the samples came from endoscopic or surgical samples of patients who had not been treated at the time of collection to avoid mutational effects; iii) the patients did not have a known syndromic or first-degree family history of CRC to focus on presumed sporadic CRC; iv) the tumors were micro-satellite stable (MSS); v) the patients were Saudi; this was done to include Saudi patients; and vi) there was enough tumor DNA to match normal tissue DNA (>0.9 µg).

We compared our cohort to two other CRC WES groups: one from the MD Anderson Cancer Center (MDACC) and the Cancer Genome Atlas (TCGA) colon adenocarcinoma (COAD) and rectum adenocarcinoma (READ) (14). This was done to see if there were any ethnic differences in the genomic landscape of colorectal cancer between Saudi patients and European CRC patients. These two external cohorts had different ethnic genomic backgrounds compared to the Saudi population.

The Institutional Review Boards of MDACC and KSUMC granted approval for this study. The procedures were performed in compliance with the Declaration of Helsinki. All recruited patients provided written consent after being fully informed. **Table 1** provides a summary of.

**Table 1:** Demographic and clinical characteristics of the colorectal cancer cohort across three datasets.

Characteristic	KSU (N = 48)	MDACC (N = 131)	TCGA (N = 322)	Overall (N = 501)	P-value
Gender					
FEMALE	25 (52.1%)	59 (45.0%)	143 (44.4%)	227 (45.3%)	0.802
MALE	23 (47.9%)	72 (55.0%)	179 (55.6%)	274 (54.7%)	
Age at diagnosis (years)					
Mean (SD)	56.4 (13.2)	61.7 (12.9)	66.2 (12.2)	64.1 (12.9)	<0.001
Median [Min, Max]	58.0 [26.0, 84.0]	62.0 [29.0, 93.0]	67.0 [33.0, 90.0]	65.0 [26.0, 93.0]	
Stage					
I	2 (4.2%)	0 (0%)	10 (3.1%)	12 (2.4%)	<0.001
II	16 (33.3%)	5 (3.8%)	56 (17.4%)	77 (15.4%)	
III	24 (50.0%)	104 (79.4%)	221 (68.6%)	349 (69.7%)	
IV	6 (12.5%)	22 (16.8%)	32 (9.9%)	60 (12.0%)	
Missing	0 (0%)	0 (0%)	3 (0.9%)	3 (0.6%)	
Tumor site					
Ascending Colon	8 (16.7%)	21 (16.0%)	36 (11.2%)	65 (13.0%)	0.008
Cecum	5 (10.4%)	31 (23.7%)	50 (15.5%)	86 (17.2%)	
Descending Colon	6 (12.5%)	10 (7.6%)	9 (2.8%)	25 (5.0%)	
Rectosigmoid Junction	2 (4.2%)	5 (3.8%)	35 (10.9%)	42 (8.4%)	
Rectum	6 (12.5%)	14 (10.7%)	61 (18.9%)	81 (16.2%)	
Sigmoid Colon	20 (41.7%)	31 (23.7%)	84 (26.1%)	135 (26.9%)	
Transverse Colon	1 (2.1%)	13 (9.9%)	20 (6.2%)	34 (6.8%)	
Hepatic Flexure	0 (0%)	1 (0.8%)	15 (4.7%)	16 (3.2%)	
Splenic Flexure	0 (0%)	1 (0.8%)	5 (1.6%)	6 (1.2%)	
Others	0 (0%)	0 (0%)	7 (2.2%)	7 (1.4%)	
Missing	0 (0%)	4 (3.1%)	0 (0%)	4 (0.8%)	

### Whole-exome sequencing

Genomic DNA was extracted from fresh frozen tissue with the QIAamp DNA mini Tissue kits (Qiagen). The extracted DNA was tested for purity, amount, and integrity. The identity of each extracted DNA sample was tested using the AmpFlSTR Identifiler PCR Amplification Kit (Thermo Fisher Scientific). The Novagene sequencing lab in South Korea used the HiSeq2000 platform to do whole-exome sequencing (WES) of DNA from tumors and match normal tissues from Saudi samples. We aimed for a depth of at least 50×; the effort successfully achieved a commendable 80% coverage of mapping bases with at least 8× coverage and around 93% of the genome being sequenced. Samples from the MDACC cohort were sequenced using the sample platform with similar criteria at MDACC’s core facility. We obtained the sequencing reads of TCGA through TCGA bioportal to apply the same bioinformatic methods for all three cohorts and limit the method’s bias.

### Bioinformatics

The same bioinformatic pipeline and computational methods were applied to the three cohorts. Raw sequencing reads were aligned to the human genome (hg19) using BWA with default parameters (15). Bam files were then sorted using Sam tools and processed using GATK kit (16). Variants were then identified using Mutect2, and only variants with a minimum sequencing depth of 30 and alternate alleles supported by at least 5% of reads were chosen (17). Potential germline variants (MAF >5% in the ExAC (http://exac.broadinstitute.org/) database) were masked after annotation using ANNOVAR for functional consequence (18–20). The variant call files (VCFs), which contained annotated somatic variations, were transformed into mutation annotation format (maf) files. The Maftools R package was used to analyze and visualize the somatic profiles of all cohorts (21). The maftools were used to identify the genes that have recurring mutations. This was done by analyzing the number of samples that had mutations in their coding sequence, including UTRs. The SMGs were obtained by utilizing the maf file as the input for the MutSigCV algorithm (22). The selection of significantly mutated genes (SMGs) was using a threshold of (q < 0.01) for the false discovery rate (FDR). The somatic Interactions function in R maftools was utilized to conduct pair-wise Fisher’s exact tests. This analysis aimed to ascertain the mutation exclusivity or co-occurrence among the top 25 genes. A significance level of P < 0.05 was used. The variable allele fraction (VAF) was determined by dividing the proportion of reads seen at a particular variation by the total coverage at the variant position. The results of this calculation were presented in order to determine the clonal status of the genes that were most frequently altered. Mutations with a high variant allele frequency (VAF greater than 15%) were identified in the coding sequences of certain genes. Pathways were visualized using pathway mapper and analysis was conducted utilizing the pathway templates of TCGA (23, 24). Tumor mutational burden (TMB) is defined as the quantity of somatic mutations per megabase. Determined by dividing the total number of qualifying tumor mutations by the size of the interrogated coding region (30 Mb) and presenting the result on a logarithmic scale.

### Microsatellite instability

Immunohistochemistry (IHC) was used to confirm that all of the samples were MSS tumors, and the MS sensor algorithm was used to confirm samples that had not been tested (25). Primary monoclonal antibodies to MLH1, MSH2, MSH6, and PMS2 were utilized by IHC testing for Mismatch Repair (MMR) gene deficiency.

### Statistical analyses

All statistical analyses were performed using R v 4.1.0 (https://www.r-project.org/). Fisher’s exact test was used or categorical data, Wilcoxon rank test or Student’s t-test we used for continuous data, as needed. P-values were adjusted for multiple testing using the FDR. P < 0.05 was the significance threshold, unless otherwise stated.

## Results

### Overview of cohorts’ characteristics

**Figure 1:**
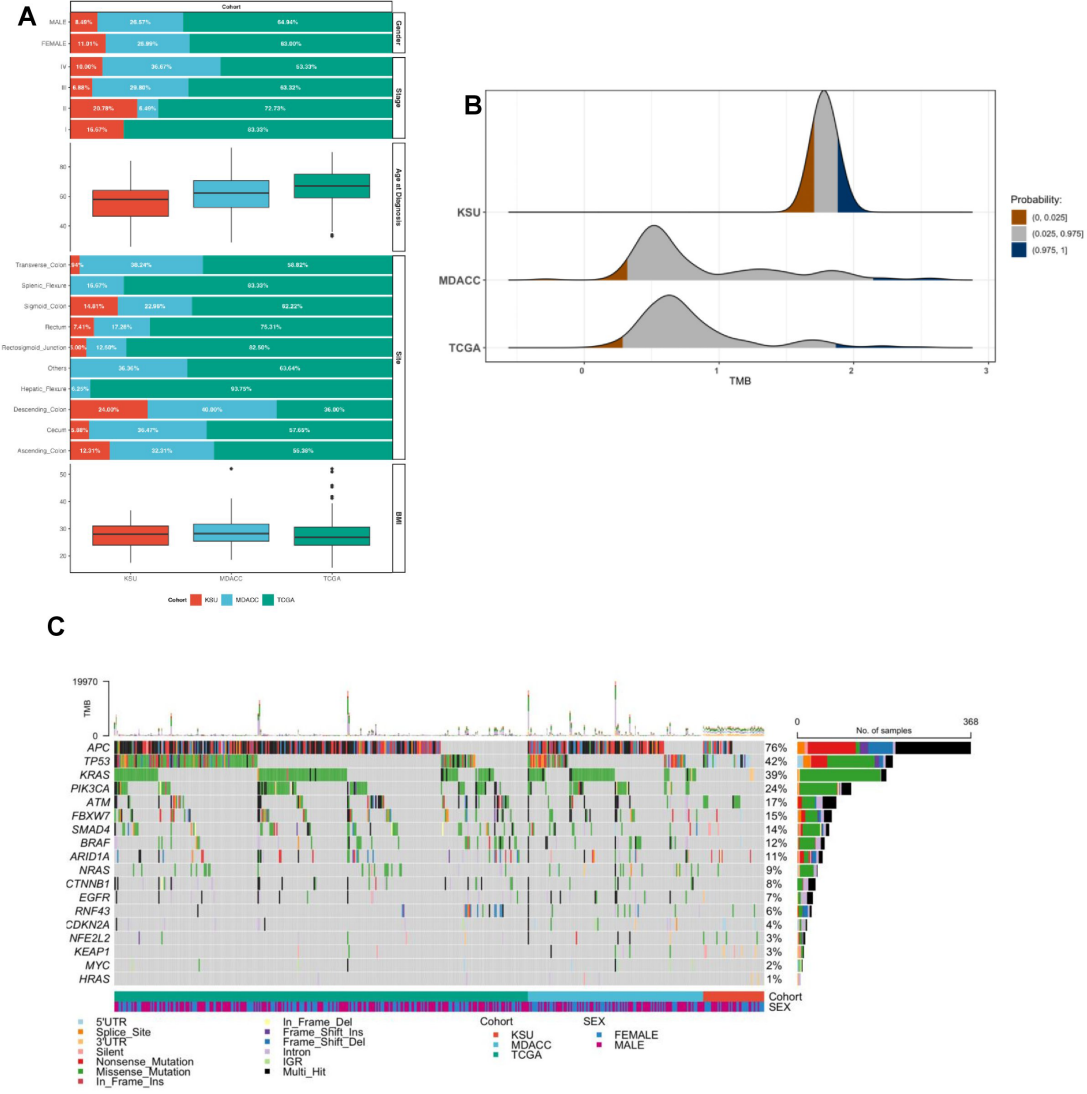
**(A)** This figure displays the distribution of various clinical and demographic characteristics across three colorectal cancer cohorts: KSU, MDACC, and TCGA. The top section of the figure shows the proportion of male and female patients, along with the distribution of cancer stages **(I-IV)** across each cohort. The middle section provides box plots representing age at diagnosis for each cohort, illustrating medians, quartiles, and outliers. The bottom section categorizes patients based on the anatomical site of the cancer, highlighting differences in site-specific prevalence across cohorts. **(B)** Comparison of log tumor mutational burden (TMB) of non-synonymous mutations/30 MB across three different datasets: KSU, MDACC, and TCGA. The x-axis represents the TMB score ranging from 0 to 3, while the y-axis indicates the probability density for each dataset. Different probability ranges are shown in color: brown for the lowest probability range (0, 0.025), gray for the middle range (0.025, 0.975), and blue for the highest probability range (0.975, 1). The figure highlights the distribution and variance of TMB scores within and between these datasets, illustrating differences in genomic characteristics of the cohorts analyzed. **(C)** Landscape of CRC driver gene somatic variants profile in colorectal cancer across different groups. Each column represents an individual patient, while each row corresponds to a specific gene. The color coding denotes different types of mutations, mutation subtypes, study groups and genders. The bar graph at the top shows the average number of mutations per patient, while the bar graph on the right displays the mutational load for each gene. The plot provides a comprehensive overview of the CRC mutational spectrum and highlights the unique genetic landscape of CRC among different populations. * p ≤ 0.05, ** p ≤ 0.01, *** p ≤ 0.001.

In total, 48 Saudi patients from King Saud University (KSU) were enrolled, 131 patients from MD Anderson Cancer Center (MDACC), and 322 patients from The Cancer Genome Atlas (TCGA) were included for comparison (**Figure 1A**; **Table 1**). Every patient from each of the three cohorts that was included had a diagnosis of sporadic microsatellite stable (MSS) adenocarcinoma of the colon or rectum. The Saudi patients were considerably younger than the MDACC and TCGA patients (62 and 66 years old, respectively), with a median age of 58 years (range: 26–84 years old) at the time of diagnosis (P < 0.001). There was a significant difference in the tumor location between the cohorts: among Saudi patients, sigmoid colon cancer was the most common anatomical site at 41.7%, compared to 23.7% in MDACC and 26.1% in TCGA (P < 0.001). The most common stage among Saudi patients was a stage III disease (50% vs. 79.4% MDA and 68.6% TCGA, P < 0.001), which differed from the other cohorts’ stages at presentation. The gender proportions were similar without any differences between the cohorts (p = 0.8), indicating that gender may not be a distinguishing factor in the incidence of MSS adenocarcinoma in these diverse cohorts.

### Colorectal cancer molecular comparison

High-depth whole-exome sequencing (WES) was performed on 50 colorectal tumors (60 × depth) and paired normal tissue (30 × depth) from Saudi patients with colorectal cancer. Two patients were excluded due to tumors not meeting the inclusion criteria, resulting in a final set of 48 Saudi patients. We performed a comprehensive somatic mutation analysis to examine the frequency of somatic mutations in driver CRC oncogenes, comparing Saudi patients with MDACC and TCGA patients. The Saudi tumors had an average of 2.0 tumor mutational burden (TMB) (mutations/Mb), which was significantly higher than the numbers for the MDACC and TCGA tumors, which were 1.08 and 1.07, respectively (P < 0.001). This difference remained significant even after adjusting for tumor stage and anatomical site (**Figure 1B**). The vast majority (90%) of the coding mutations were missense variants in all cohorts, as previously reported in CRC studies (**Figure 1C**) (14, 26).

### Colorectal cancer driver gene mutational analysis

**Figure 2:**
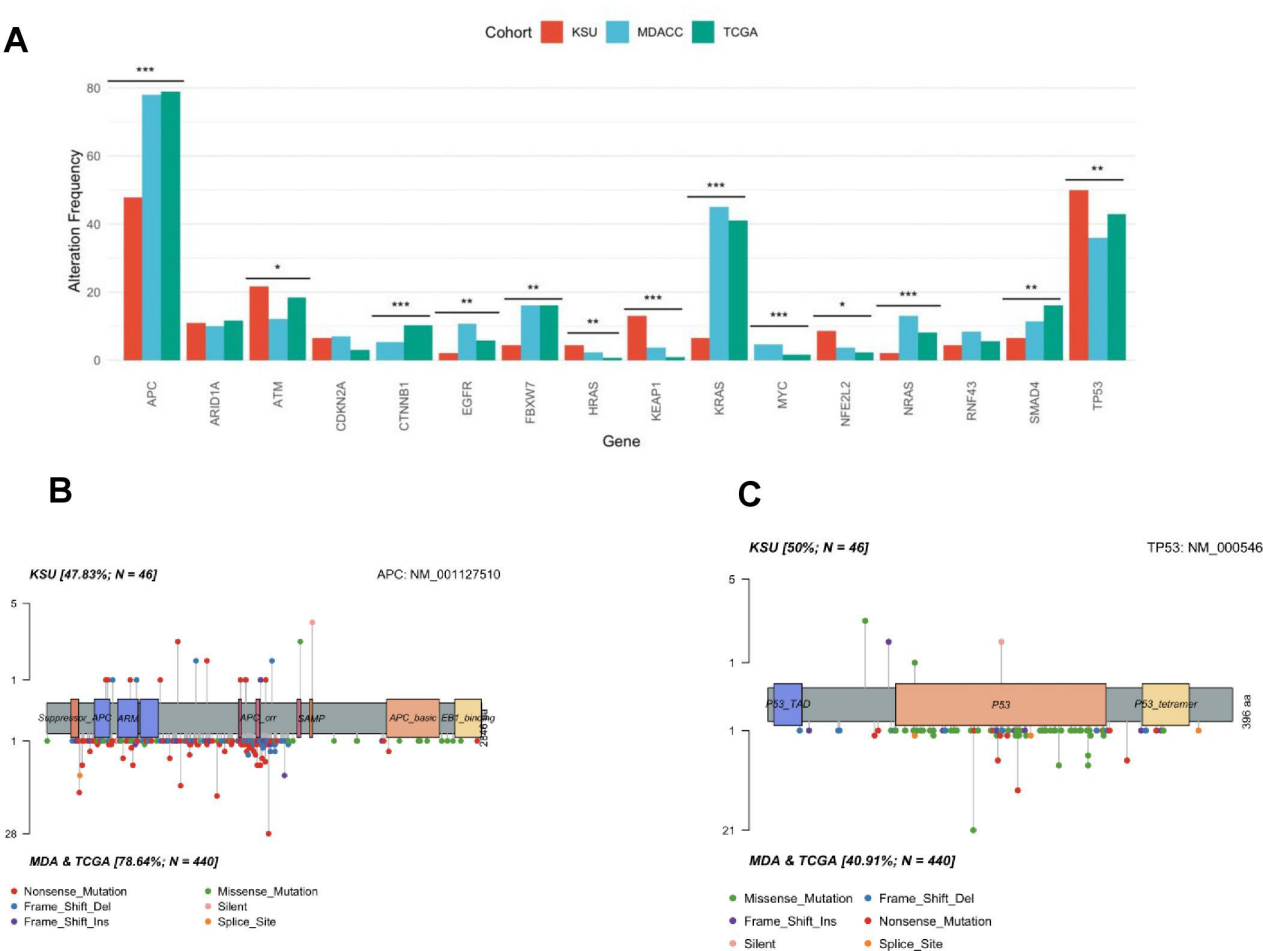
**(A)** Frequency of CRC oncogenic gene variants in Saudi patients, MDACC and TCGA patients compared. **(B)** The lollipop plots of somatic mutational variants in the *APC* gene comparing the Saudi tumors on top with MDACC and TCGA on the bottom. **(C)** The lollipop plots of somatic mutational variants in the *TP53* gene comparing the Saudi tumors on top with MDACC and TCGA on the bottom. * p ≤ 0.05, ** p ≤ 0.01, *** p ≤ 0.001.

We analyzed the known CRC driver gene differences among the cohorts. We identified significant differences in frequency between some CRC genes. The *APC* gene was mutated in 76% of all patients (**Figure 1C**). However, the frequency of APC mutations was significantly lower in the Saudi cohort at 47.8% compared to 77.9% in MDACC and 79% in TCGA, indicating a notable regional variation in genetic profiles (p < 0.001) (**Figures 2A, B**). Conversely, *TP53* mutations were more prevalent in the Saudi cohort at 50%, significantly higher than MDACC’s 35.6% suggesting a potential distinctive pathway of tumorigenesis in this population (**Figure 2C**). Additionally, mutations in KRAS and BRAF were notably less frequent in the Saudi samples at 6.5% and 2.1%, respectively (**Figure 2A**). The prevalence of mutations in other commonly changed oncogenes involved in CRC carcinogenesis did not differ across the Saudi tumors, MDACC, and TCGA.

### Colorectal cancer signaling pathway analysis

**Figure 3:**
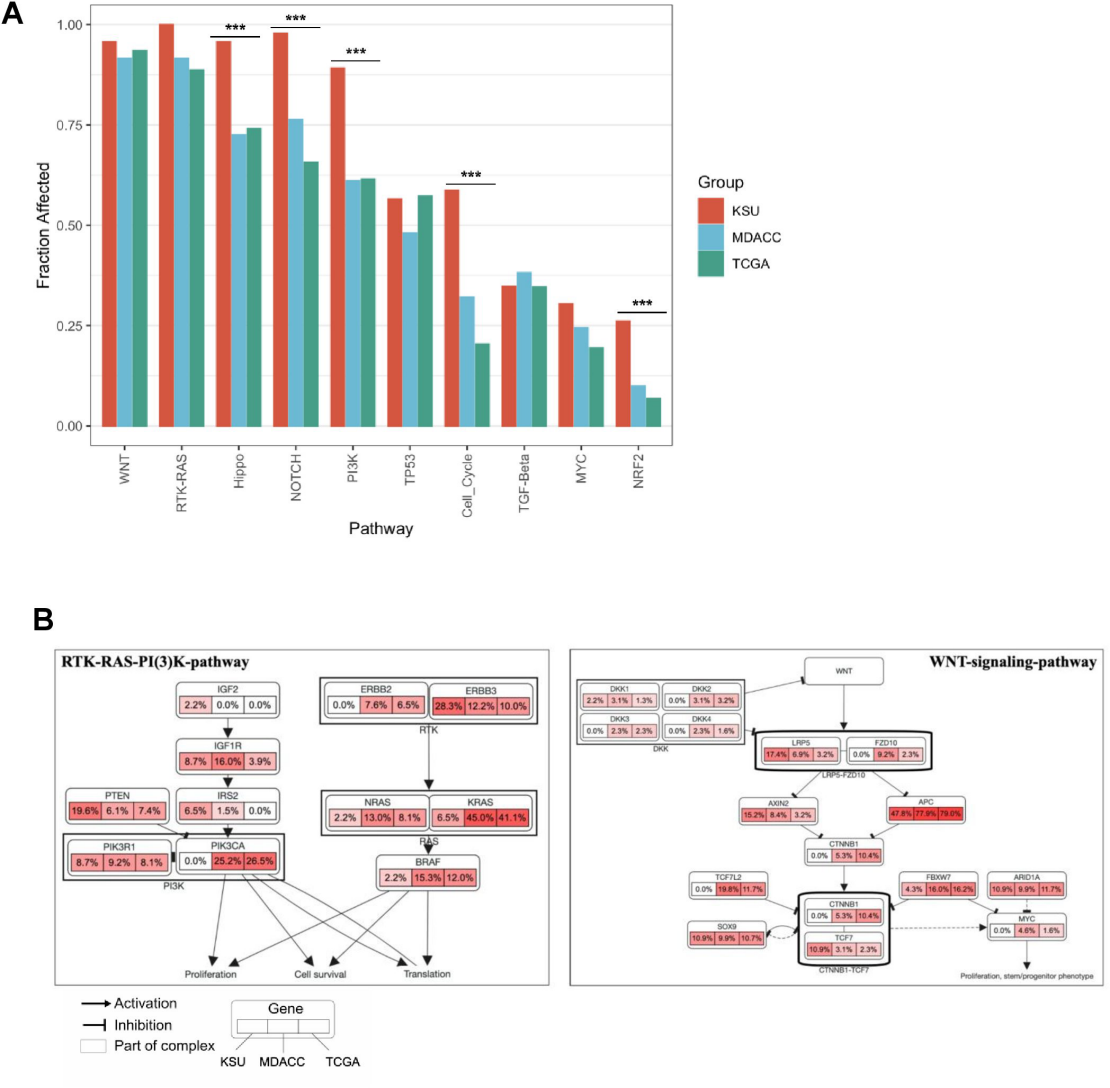
**(A)** Frequency of CRC canonical signaling pathways in Saudi patients, MDACC, and TCGA patients compared. **(B)** Specific genomic alterations and frequencies within the RTK/RAS AND WNT oncogenic pathways stratified by the Saudi MDACC and TCGA tumors, respectively.

By CRC canonical signaling pathways, somatic mutations were further divided (**Figure 3A**). Although WNT signaling pathway genes showed similar significant variances (*CTNNB1, FBXW7, TCG7* and *DKK* genes) in prevalence in Saudi CRC cancers, the overall alteration frequency of WNT signaling pathway did not differ between Saudi and MDACC patients, indicating possible distinct activation of the WNT signaling system while illustrating its central role in colorectal cancer (**Figures 2A**, **3A**). Alterations within the WNT and RAS pathways in all patients are presented in (**Figure 3B**). The Saudi group also had more changes in the Hippo, Notch, PI3K, cell cycle, and NRF2 pathways compared to the MDACC and TCGA groups (**Figure 1**). This suggests that Saudi CRC tumors play a different role.

The findings indicate a unique CRC mutational profile in the Saudi population emphasizing the need for population-specific studies to enhance CRC diagnostic, screening, and treatment strategies in Saudi Arabia.

## Discussion

Colorectal cancer is highly genetically heterogeneous disease with an alarming prevalence in Saudi Arabia, particularly in men and at earlier onset (1–3). However, there has not been any whole-exome sequencing investigating the Saudi CRC somatic mutational landscape. Here, in a sporadic MSS CRC Saudi cohort, we describe the somatic events underpinning CRC leveraging deep whole-exome sequencing approach. We then compared the findings to an independent cohort from MDACC in addition to TCGA data.

In contrast to the classical Vogelstein adenoma–carcinoma sequence, the Saudi CRC cohort shows lower *APC*, *KRAS*, and *BRAF* mutation frequencies together with a higher prevalence of *TP53* alterations, pointing towards a distinct mutational signature that may be shaped by genetic, environmental, or lifestyle factors specific to this population. The reduced mutation rates in genes well-established in CRC oncogenesis globally, juxtaposed with the younger median age at diagnosis and higher prevalence of rectosigmoid disease, underscore the necessity for a tailored approach in understanding CRC in Saudi Arabia.

The substantial discrepancy in mutation rates of key driver genes warrants a deeper delve into the genetic and environmental factors that may contribute to the distinct mutational landscape observed. It also raises questions on whether the current global therapeutic strategies and diagnostic markers are fully applicable to the Saudi population or if there’s a need for region-specific adaptations. Furthermore, the distinctive genomic signature could potentially harbor novel therapeutic targets, underscoring the need for continued research on the genomic of CRC in Saudi Arabia.

Our comparative analysis with TCGA and MDACC cohorts illuminates the genetic heterogeneity inherent in Saudi CRC, reaffirming the importance of population-specific studies in decoding the complex genomic architecture of this malignancy. The findings also accentuate the imperative for enhanced regional genomic databases and collaborative research endeavors to foster a more nuanced understanding of CRC, which in turn, can significantly impact the diagnostic and therapeutic paradigms in the Saudi Arabia.

The conservative genetic pool of KSA, characterized by high rates of consanguinity, offers a distinctive opportunity to study CRC through the lens of genetic predispositions that may differ significantly from those observed in more genetically diverse populations. The high rate of genetic homogeneity could lead to the prevalence of specific mutations and genetic patterns that are rare elsewhere, providing a unique model for studying the genetics of CRC and its impact on disease manifestation and treatment outcomes. Additionally, extended runs of homozygosity (ROH) are likely to be more frequent potentially enriching for recessive risk variants and population-specific genomic architectures that could influence CRC susceptibility, clinical presentation, and response to treatment (27, 28).

The comprehensive mutational analysis conducted in this study provides a stepping stone towards a more profound understanding of CRC in Saudi Arabia, while acknowledging that the relatively small cohort size limits statistical power, particularly for low-frequency events. Building on this initial dataset, future larger studies are needed to validate these findings and further decipher the molecular mechanisms driving CRC in this population, ultimately informing personalized therapeutic strategies and improving CRC management in Saudi Arabia and in similar genetic or environmental settings.

Moreover, the collaboration with established institutions and utilization of advanced sequencing and bioinformatics tools have been instrumental in dissecting the mutational landscape of CRC in the Saudi population. Future endeavors should focus on expanding the cohort size, exploring the impact of lifestyle and environmental factors, and establishing a comprehensive regional cancer genomics database to further elucidate the unique genomic landscape of CRC in Saudi Arabia and its implications on patient care.

## Data Availability

The original contributions presented in the study are included in the article/supplementary material. Further inquiries can be directed to the corresponding author.
